# Mapping complex traits using Random Forests

**DOI:** 10.1186/1471-2156-4-S1-S64

**Published:** 2003-12-31

**Authors:** Alexandre Bureau, Josée Dupuis, Brooke Hayward, Kathleen Falls, Paul Van Eerdewegh

**Affiliations:** 1 Genome Therapeutics Corporation, Waltham, Massachusetts, 02453, USA; 2 Current address: School of Health Sciences, University of Lethbridge, Lethbridge, Alberta, T1K 3M4, Canada; 3 Current address: Department of Biostatistics, Boston University, Boston, Massachusetts, 02215, USA; 4 Department of Psychiatry, Harvard Medical School, Boston, Massachusetts, 02115, USA

## Abstract

Random Forest is a prediction technique based on growing trees on bootstrap samples of data, in conjunction with a random selection of explanatory variables to define the best split at each node. In the case of a quantitative outcome, the tree predictor takes on a numerical value. We applied Random Forest to the first replicate of the Genetic Analysis Workshop 13 simulated data set, with the sibling pairs as our units of analysis and identity by descent (IBD) at selected loci as our explanatory variables. With the knowledge of the true model, we performed two sets of analyses on three phenotypes: HDL, triglycerides, and glucose. The goal was to approach the mapping of complex traits from a multivariate perspective. The first set of analyses mimics a candidate gene approach with a high proportion of true genes among the predictors while the second set represents a genome scan analysis using microsatellite markers. Random Forest was able to identify a few of the major genes influencing the phenotypes, such as baseline HDL and triglycerides, but failed to identify the major genes regulating baseline glucose levels.

## Background

Trees are nonparametric prediction models. In the context of genetic linkage analysis, Zhang et al. [1] used single trees to identify markers where mean identity-by-descent (IBD) sharing in sib pairs is predictive of the affection status of the pair. Breiman [2] and others have reported that significant improvements in prediction accuracy are achieved by using a collection of trees, called a random forest. We applied Random Forest to the first replicate of the Genetic Analysis Workshop 13 simulated data set, with the sibling pairs as our units of analysis. We used the IBD scores (mean IBD or probability of sharing two alleles IBD) at various loci on the genome to predict the absolute difference between phenotypic values in each pair of siblings. In the candidate gene approach, IBD was estimated at the location of the candidates while in the genome scan approach, IBD was harvested at each microsatellite marker.

## Methods

We used MEGA2 [3] to create all nuclear families from the data set and then computed multipoint IBD probabilities on the whole genome for each family with GENEHUNTER [4]. We created two variables out of the IBD sharing probabilities: mean IBD and probability of sharing two alleles (Z_2_). We performed separate analyses using the mean IBD only and Z_2 _only, and a joint analysis using both mean IBD and Z_2 _as explanatory variables.

We focused our efforts on baseline levels of the following selection of traits: fasting glucose levels, fasting triglyceride level, and HDL level. Baseline values for each individual were estimated using the first visit measurement for glucose and triglyceride levels, because those traits increase with age. HDL level is stable over time and we used the mean HDL level over all visits to get a more precise estimate. These variables were stratified by ever having smoked and gender and then adjusted for covariates using the SAS [5] software program PROC REG. The covariates used to adjust the final variables were body mass index (BMI) (mean over all visits), baseline grams of alcohol per day, smoking amount (baseline number of cigarettes per day), and baseline age. In adjusting for smoking, both linear and quadratic terms were included. Plots of the adjusted trait values against the covariates showed no remaining trend. We then computed the absolute difference between the values of these adjusted variables for each sibling pair. A fourth phenotype was considered: the first principal component, as calculated by PROC PRINCOMP in SAS, of the absolute sib-pair differences of the adjusted glucose, adjusted triglycerides, and adjusted HDL levels. The first principal component explains 38% of the variance of the three phenotype differences.

A Random Forest is created by growing trees, without pruning, on bootstrap samples of the data, selecting the best split at each node among a random selection of the explanatory variables. For quantitative outcomes the forest is made of regression trees, where the tree predictor is the mean value of the training set observations in each terminal leaf. The Random Forest predictor is computed by averaging the tree predictors over trees for which the given observation was "out-of-the bag", i.e., not included in the bootstrap sample used to build the tree. The mean squared generalization error or predictive error (PE) is used to assess the predictive accuracy. Because the prediction for an observation is based on trees grown without the observation, an idea akin to cross-validation, the estimated PEs are unbiased. The importance of a variable is quantified by calculating the PE increase resulting from randomly permuting the values of a particular variable among the out-of-bag observations for each tree. Shuffling the values of variables of high importance should increase the PE, while the PE should not be affected by permuting values of variables with low importance. Random Forest regression was performed using the program RRFOREST [6] with the following parameter settings: the number of variables selected at each node was set to 30 in the candidate gene approach and to 100 in the genome-scan setting, roughly one-fourth of the total number of variables. The number of trees was set to a value where empirical evidence indicated that the importance index estimates had converged. That value was 5000 in the candidate gene analysis and 10,000 in the genome scan.

The set of candidate genes consisted of all quantitative trait loci (QTLs) affecting clinical phenotypes (b12 to b38 and s3 to s12) and four random locations on each of chromosomes 2, 6, 10, and 16, which contain no QTL [7]. The 399 marker loci were included in the genome-scan analysis. Since IBD is measured at marker loci instead of directly at the QTLs in the genome-scan analysis, a definition of a region surrounding marker loci with high importance index values is needed to determine whether an important marker locus is close enough to a QTL to be called a true positive. We defined an importance peak interval as a region surrounding one or more consecutive markers with importance greater than 0.05%. Each peak interval originates at a marker that is a local maximum of the importance index and extends to the left and right over 10 cM or to the first marker with importance lower than 0.05%, whichever is furthest away. Overlapping peak intervals were collapsed into a single peak interval.

We combined the results for the separate mean IBD and Z_2 _analyses by ranking the peak intervals from the two analyses in decreasing order of the maximum importance reached within the interval, counting overlapping peak intervals only once. The success of that combined analysis was evaluated by determining whether each peak interval contained genes influencing the particular trait. Candidate genes were ranked similarly.

## Results

**Figure 1:**
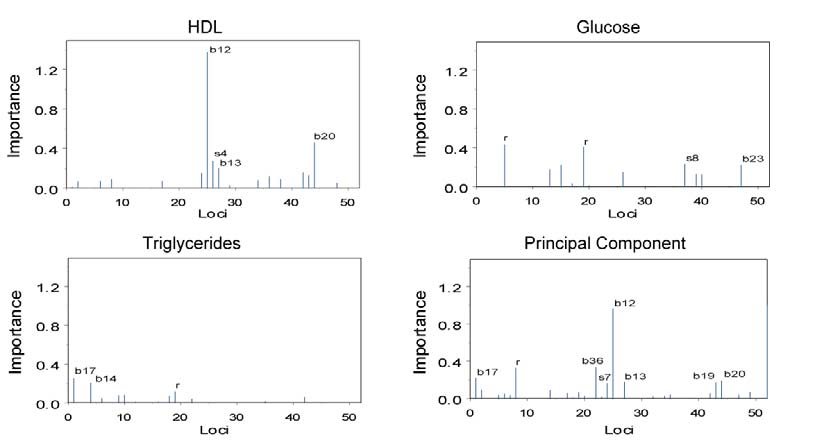
**Importance of candidate genes **Importance measured by percent increase in PE. Variables are the mean IBD at genes b12 to b38 and s3 to s12 and at four random locations (r) on each of chromosomes 2, 6, 10, and 16 (total of 52 loci, b37 and s12 being merged into one). Random forests are 5000 trees. A sample of 30 variables is considered at each split.

**Figure 2:**
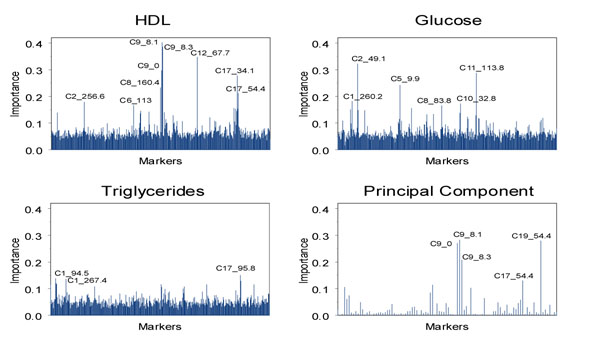
**Importance of genome scan markers **Importance measured by percent increase in PE. Variables are the mean IBD at the 399 genome scan markers. Only the variables with non-zero importance are shown. Random forests are 10,000 trees. A sample of 100 variables is considered at each split.

**Figure 3:**
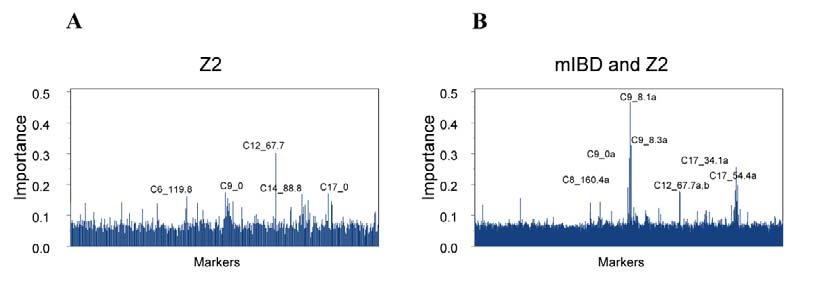
**Importance of genome scan markers using Z_2 _and both mean IBD and Z_2 _for HDL level **Importance of Z_2 _at the 399 genome scan markers (A). Importance of mean IBD and Z_2 _at the 399 genome scan markers (798 variables) (B). Only the variables with non-zero importance are shown. Random forests are 10,000 trees. A sample of 100 variables is considered at each split.

The importance of the mean IBD at candidate genes for predicting phenotype absolute differences is reported in Figure 1. The same measure at the genome scan markers is reported in Figure 2. For adjusted HDL level difference, genes b12 and b20, which both contribute to the HDL phenotype, have the highest importance in the candidate gene analysis. Mean IBD at the first three markers on chromosome 9, close to b12, at a random location on chromosome 12, and at two locations on chromosome 17 linked to genes b19, b20, b18, and b29 have the highest importance in the genome-scan analysis. Figure 3 shows the importance values when using Z_2 _and both mean IBD and Z_2 _(joint analysis) in the genome-scan analysis. While the region around b12 on chromosome 9 is identified by all choices of IBD measures, the chromosome 17 region is common only to the mean IBD and joint analyses. The Z_2 _analysis points to a chromosome 17 locus near s11 that is not seen in the other two analyses. In general, we observed more consistency between the mean IBD and joint analyses than between those two types of analysis and the Z_2 _analysis. For adjusted triglyceride level difference, the mean IBD at candidate genes b17 and b14 is the highest. A marker on chromosome 1 linked to b17 is detected in the genome scan analysis, as well as a false-positive signal on chromosome 17. For adjusted glucose level difference, the candidate genes most predictive of the phenotype were random locations on even numbered chromosomes. Similarly, the locations with the highest importance in the genome scan are unlinked to any susceptibility gene. In the analysis of the first principal component of the adjusted HDL, triglyceride, and glucose level differences, the mean IBD at gene b12 stands out as the most important in both analyses. The second highest importance peak in the genome scan analysis on chromosome 19 does not contain any QTL.

The ranking of the importance peaks from combining the mean IBD and Z_2 _separate analyses in Table 1 shows QTLs among the top hits with both candidate genes and genome scan markers for all phenotypes except glucose level. A few genes influencing a phenotype indirectly were also detected. The candidate gene analysis yielded fewer false positives, as expected.

## Discussion

Although Random Forest was able to identify several genes in the candidate-gene approach and several chromosomal regions harboring susceptibility genes in the genome-scan approach, the findings were not consistent across different phenotypes. In addition, the ranking of genes identified did not typically follow the ranking of effect sizes used in the simulation. The predictive power was in general very low, with PE in excess of 98% of the variance of the absolute difference. However, that observation does not imply failure in the context of a linkage analysis. By analogy, the Haseman and Elston [8] regression of squared difference against IBD sharing in sibs may give a very significant result at a disease locus while at the same time explain little of the total variance of the regressed variable. A measure of the variability of the importance index would be more relevant to assess the significance of observed importance levels.

Since the selected phenotypes are correlated, some genes also have an indirect influence on phenotypes other than the one they directly affect. Genes affecting phenotypes correlated to the one analyzed do figure among the loci with highest importance in Table 1 and could represent indirect effects. However, we did not expect the genes influencing the slope with age to show elevated importance for baseline levels.

We elected to use the baseline value of the phenotypes considered because a larger number of genes were affecting baseline value than the slope of the phenotype with age. Since we did not identify any baseline genes for glucose levels in our first round of analyses, we subsequently applied the Random Forest method to the slope of the regression of glucose level against age within individual after some groups reported extremely strong signals near the s3 gene on chromosome 5. We observed importance levels much larger than those reported in Figures 1 and 2 (increases in PE exceeding 5% at b22, a locus tightly linked to s3, and around 3% at s3 itself, but no other candidate above 0.5%, under the candidate gene approach, and exceeding 0.8% at the closest marker to s3 with no other peak reaching 0.3% in the genome scan analysis, using either mean IBD or Z_2_). This confirms that Random Forest can identify a major locus when one is present.

It is encouraging that the ability to detect genes was not limited to the candidate gene setting which had relatively few random locations unlinked to each of the three phenotypes. In the genome-scan approach where we simultaneously considered all genome scan markers, Random Forest was again able to select several genomic regions of interest without substantially increasing the number of false-positive signals among variables with the largest importance, compared to the candidate-gene approach.

## Figures and Tables

**Table 1:** QTLs at candidate loci and in peak intervals with highest importance

	**Candidate Genes Analysis**				**Genome Scan Analysis**			
		
**Rank**	**HDL**	**Glucose**	**Triglycerides**	**1^st ^Principal Component**	**HDL**	**Glucose**	**Triglycerides**	**1^st ^Principal Component**
1	b12	FP^B^	b17	b12	b12	FP	FP	b19,b20
2	b20	FP	b14	b20	FP	FP	FP	b13
3	b13	FP	b12	FP	b19,b20,b18	b30^A^	b13	b12
4	s3^A^	FP	b27^A^	FP	FP	s3	b14	FP
5	s4^A^	FP	FP	b17	FP	FP	FP	FP
6	FP	FP	FP	b19	FP	FP	b17	FP
7	b19	FP	b33^A^	b20	FP	b14	FP	b29
8	FP	b23^A^	b25	FP	FP	FP	b12	b22, s3
9	FP	b22^A^	FP	b13	FP	FP	FP	FP
10	s7^A^	b18	FP	FP	b24^A^	FP	b35^A^	FP
11	b24^A^	FP	FP	s7^A^	b26^A^	FP	FP	FP
12	FP	s4	FP	s3	b20	FP	FP	b14

List of genes influencing a trait directly or indirectly through a correlated trait. For peak intervals (genome scan), all genes directly affecting a trait are listed. ^A^ Genes influencing only a correlated trait. ^B^FP, false positive (for genome scan, peak intervals containing no gene influencing the trait).
